# Patterns of Failure after Radical Surgery among Patients with Thoracic Esophageal Squamous Cell Carcinoma: Implications for the Clinical Target Volume Design of Postoperative Radiotherapy

**DOI:** 10.1371/journal.pone.0097225

**Published:** 2014-05-12

**Authors:** Qi Liu, Xu-Wei Cai, Bin Wu, Zheng-Fei Zhu, Hai-Quan Chen, Xiao-Long Fu

**Affiliations:** 1 Department of Radiation Oncology, Fudan University Shanghai Cancer Center, Shanghai, P.R. China; 2 Department of Oncology, Shanghai Medical College, Fudan University, Shanghai, P.R. China; 3 Department of Radiology, Fudan University Shanghai Cancer Center, Shanghai, P.R. China; 4 Department of Thoracic Surgery, Fudan University Shanghai Cancer Center, Shanghai, P.R. China; West German Cancer Center, Germany

## Abstract

**Background:**

This study evaluated patterns of treatment failure (especially locoregional failure; LRF) after radical esophagectomy and proposes a clinical target volume (CTV) for postoperative radiotherapy (PORT) among patients with thoracic esophageal squamous cell carcinoma (SCC).

**Methods:**

All patients who were followed up in our center after radical esophagectomy between 2007 and 2011 were retrospectively enrolled. The patterns of first discovered failure were assessed, and LRFs (including anastomotic and regional lymph node recurrences) were evaluated to determine whether our proposed PORT CTV encompassed these areas. The clinicopathologic factors predictive of lymphatic recurrence type were analyzed.

**Results:**

Of the 414 patients who underwent surgery and were followed up over the study, 207 experienced recurrent or metastatic diseases. The median time to progression was 11.0 months. Of the 173 patients with locoregional recurrence, nodal failure recurred in 160; supraclavicular and superior mediastinal lymph nodes had the highest metastasis rates. All 233 recurrent sites across the 160 patients were located in a standard CTV area, including the bilateral supraclavicular areas, the entire mediastinum, and the left gastric lymphatic drainage region. A total of 203 sites (87.2%) were located in either the bilateral supraclavicular areas or the entire mediastinum, and 185 sites (79.4%) were located in either the bilateral supraclavicular areas or the upper mediastinum. A multivariate analysis revealed the lymph node metastatic ratio (LNMR) and tumor differentiation were risk factors for nodal failure.

**Conclusions:**

Locoregional recurrence (especially lymph node recurrence) was the most common and potentially preventable type of initial treatment failure after curative surgery among patients with thoracic esophageal SCC. The proposed PORT CTV covered most LRF sites. The lymphatic drainage regions for PORT are selective, and the supraclavicular and superior mediastinal areas should be considered. However, the value of PORT and the extent of CTV should be investigated in further prospective studies.

## Introduction

Surgery is the most important initial treatment for patients with thoracic esophageal squamous cell carcinoma (SCC). However, the recurrence rate of SCC is as high as 40%–50% after radical surgery [1], and locoregional recurrence is the major cause of treatment failure [2], [3], even among patients with a pathologically complete response to neoadjuvant chemoradiotherapy [4]. van Hagen et al. [5] indicated that overall survival (OS) and local tumor control could be improved using neoadjuvant chemoradiotherapy, which is already used at many institutions. This standard suggests that postoperative radiotherapy (PORT) should not play an important role in SCC treatment. However, SCC comprises more than 90% of the esophageal cancer cases in East Asia, and tumors located in the upper and middle thoracic esophagus are most commonly observed. In these cases, neoadjuvant radiotherapy often increases the difficulties associated with surgery due to tissue edema and hemorrhage. In addition, patients in China generally prefer surgery to neoadjuvant chemoradiation as their initial treatment. Therefore, evaluating the efficacy of adjuvant radiotherapy is essential. To date, no randomized trial has evaluated the survival advantages of PORT alone; thus, adjuvant radiotherapy is not currently recommended in the National Comprehensive Cancer Network (NCCN) treatment guidelines. According to multiple retrospective analyses, the addition of postoperative chemoradiotherapy has been associated with survival benefits among patients with locoregional esophageal carcinoma [6]. Xiao et al. [7] reported that PORT improves the survival rates of patients with positive lymph nodes and reduces the incidences of intrathoracic recurrence and supraclavicular lymph node metastasis among all patients. Chen et al. [8] retrospectively analyzed 945 patients and found similar results. Xu et al. [9] retrospectively analyzed 725 patients and reported an association between improved OS and PORT (36.6%–43.6%, p = 0.018) among patients with lymph nodes positive for stage III ESCC. A large, population-based review using the Surveillance Epidemiology and End Results database also supported the use of postoperative radiation for stage III SCC and adenocarcinoma of the esophagus [10]. PORT should be strongly considered for certain patients with esophageal SCC; however, selecting patients for adjuvant radiotherapy (RT) can be problematic. In addition, the appropriate clinical target volume (CTV) for prophylactic RT is generally disputed, particularly with regard to the extent of the lymphatic drainage region based on axial image scans.

Increased knowledge of the patterns of recurrence and metastasis after radical surgery would help to determine the irradiation targets for PORT. Accurate recurrence locations based on CT images can provide more information when contouring target volume.

This retrospective study analyzed the recurrence and metastases of thoracic esophageal SCC after radical resection based on CT scans to evaluate the risk factors that influence its recurrence patterns and provides a reference to determine appropriate PORT.

## Methods

### Patients

To be included in this study, patients must have met the following criteria: (1) radical R0 resection (complete removal of the entire tumor with clear histological margins) to treat esophageal SCC confirmed by pathological findings; (2) pathological stage T1-4aN0-3M0; (3) no prior therapy or PORT; and (4) initial regional recurrence identified using routine computed tomography (CT) scanning during the follow-up period.

The exclusion criteria were (1) a histological diagnosis of adenocarcinoma or another histological type; (2) an esophagectomy with a one-field lymphadenectomy or non-lymphadenectomy; (3) fewer than 12 removed lymph nodes; and (4) previous malignancies. Patients were postoperatively staged according to the AJCC/UICC TNM staging system (Version 7.0, 2009).

The Ethics Committee of Fudan University Shanghai Cancer Center approved this retrospective study. All patients provided written informed consent for inclusion.

### Follow up

Follow-up occurred every 3 months for the first 2 years after surgery and every 6 months thereafter. Re-examinations included cervical ultrasounds, chest enhanced CT scans, abdominal ultrasound screening, and, when necessary, bone emission computed tomography (ECT), positron emission tomography (PET)/CT, and endoscopy. Cervical and abdominal CT was required when ultrasound indicated suspicious nodes.

### Patterns of failure

**Figure 1:**
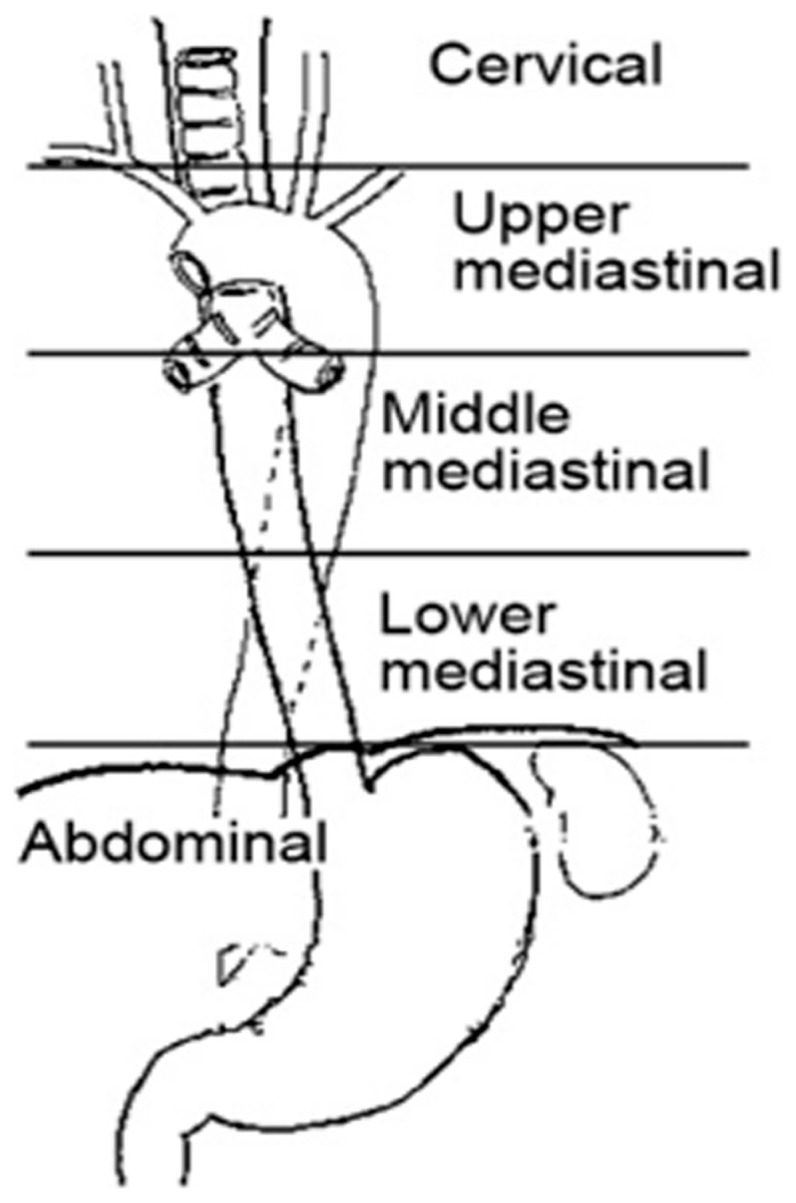
Definition of the lymph node regions.

Locoregional failures (LRFs) included esophageal and regional lymph node recurrences, which were diagnosed based on CT or PET/CT images. Suspected esophageal recurrences and neck/supraclavicular node recurrences were confirmed using histologic or cytologic testing when possible. Consistent with the staging system released by the Japanese Society for Esophageal Disease [11], the lymphatic drainage region was divided into the following five groups: cervical, upper mediastinal, middle mediastinal, lower mediastinal, and upper abdominal (Figure 1). Regional lymph node recurrence was diagnosed when (1) nodes reappeared after complete disappearance or (2) new nodes appeared in regions where enlarged nodes had not existed before. When evaluating regional lymph nodes using CT images, a short axis greater than 1.0 cm was considered positive in the transverse plane [12]. Node location was defined as the position of the center point of the largest cross section of the lymph node based on the axial CT images (confluent lymph nodes were counted as one.). Limited node recurrence was defined as single-station nodal recurrence or multi-station lymph nodes that were located in one drainage region or two adjacent regions. Extensive node recurrence was defined as multi-station recurrent nodes with an interval of at least two nonadjacent drainage regions between the highest and lowest groups of recurrent nodes.

Distant metastasis was defined as recurrent diseases appearing in other organs or non-regional lymph nodes metastasis. For those with recurrences in multiple sites, all lesions were analyzed simultaneously.

### Endpoint and analysis

The first discovered recurrence after esophagectomy was defined as the study endpoint. Disease-free survival (DFS) was defined as the elapsed time between the first diagnosis and the first relapse (local recurrence or distant metastasis). The patterns of lymphatic recurrence were analyzed. The percentage of patients for whom all sites of lymphatic recurrence would have been covered was computed as the number and percentage of failure sites within or outside the proposed PORT CTV. Clinical features are reported as the mean±standard deviation or percentage. A multivariate analysis using the Cox regression model was performed to evaluate the risk factors related to nodal recurrences. The variables in the analysis included gender, age, tumor location, pathologic T-stage, pathologic N-stage, lymph node metastatic ratio (LNMR), tumor differentiation, surgical approach and adjuvant chemotherapy. Statistical Package for Social Sciences (SPSS, version 13.0) was used for all data analyses. All tests were two sided, *P*-values less than 0.05 were considered significant.

## Results

Our hospital stored the follow-up records of 414 patients who were treated with radical surgery (but not PORT) between Jan 2007 and Dec 2011. These included 360 male patients and 54 female patients with a median age of 58 years (range = 32–77 years). 48.1% of the entire group, which included 4.9% of stage I, 41.7% of stage II, and 77.7% of stage III patients, received adjuvant chemotherapy with a regimen of fluoropyrimidine and cisplatin or carboplatin. The patients' clinical and pathological characteristics are shown in Table 1.

**Table 1:** Clinical and pathological characteristics.

Characteristics	Subcategory	No. of patients	Constituent ratio (%)
Age	Median	58	
	Range	32–77	
Gender	Male	360	87.0
	Female	54	13.0
Tumor differentiation	Well	51	12.3
	Moderately	254	61.4
	Poorly	109	26.3
pT stage	T1–2	213	51.4
	T3–4	201	48.6
pN stage	N0	210	50.7
	N1	123	29.7
	N2	56	13.5
	N3	25	6.1
Location of tumor	Upper thoracic	31	7.5
	Middle thoracic	184	44.4
	Lower thoracic	199	48.1
Lymphadenectomy	Three-field	72	17.4
	Two-field	342	82.6
Lymph nodes dissected	Median	24	
	Range	12–73	
Adjuvant chemotherapy	No	215	51.9
	Yes	199	48.1

The median follow-up time was 26 months (range = 1–77 month) for all living patients. The 1-, 2-, and 5-year DFS rates were 70.9%, 50.6%, and 27.0%, respectively. A total of 207 patients were diagnosed with recurrent, metastatic, or both types of disease. Their median time to progression was 11 months (range = 1–65 months). The most common pattern of failure was locoregional recurrence, which was found in 173 patients (83.6%). Distant failures occurred among 49 patients (23.7%); distant visceral metastasis alone occurred among 34 patients (16.4%), and both locoregional and distant failures among 15 patients (7.2%).

Of the 173 patients in which LRF sites were known, 13 were anastomotic recurrences alone, eight patients had comorbid anastomotic and lymphatic recurrences, and 15 developed both distant failures and lymphatic recurrences. Of the 160 patients with lymph node recurrences, 152 had limited lymph node recurrences (including 118 single-station lymph node recurrences and 34 limited multi-station lymph node diseases), and eight had extensive multi-station lymph node recurrences. Neck/supraclavicular, mediastinal, and upper abdominal node disease occurred among 61, 97, and 26 patients, respectively. Mediastinal nodes were primarily located in the upper and middle mediastinum, usually at the paratracheal station above the aortic arch or below the carina. The distribution of lymphatic recurrence is shown in Table 2.

**Table 2:** The distribution of LRFs.

Category	No. of patients (%)	
Cer.	43	(24.9)
Med.	77	(44.5)
Abd.	18	(10.4)
Cer.+Med.	14	(8.1)
Med.+Abd.	4	(2.3)
Cer.+Abd.	2	(1.2)
Cer.+Med.+Abd.	2	(1.2)
Ana.	13	(7.5)
Total	173	(100)

Cer.:cervical nodes; Med.:mediastinal nodes; Abd.:upper abdominal nodes; Ana.:anastomotic recurrences.

**Figure 2:**
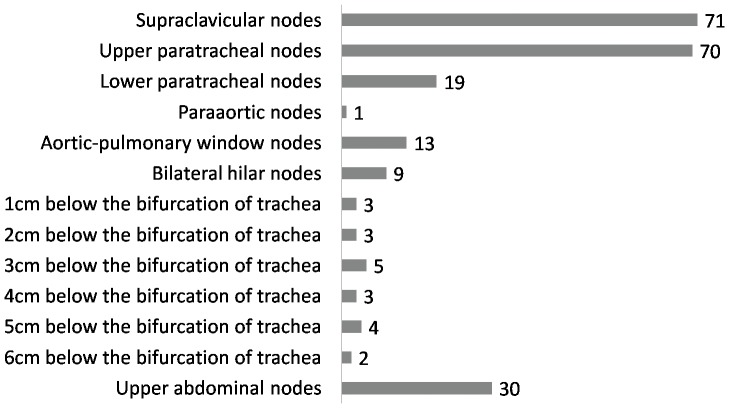
The number of recurrent sites located based on CT landmarks.

The number of recurrent sites located at CT-based landmarks is shown in Figure 2. All 233 recurrent sites among the 160 patients were located in a standard CTV area, including the bilateral supraclavicular areas, the entire mediastinum, and the left gastric lymphatic drainage region. A total of 203 sites (87.2%) were located in the bilateral supraclavicular areas and the entire mediastinum (T-shaped field); thus, the portal size was adequate for 134 of 160 patients (83.8%). If the extent of irradiation had covered the bilateral supraclavicular areas and the upper portion of the mediastinum, then 185 sites (79.4%) across 122 patients (76.3%) would have been within the area.

A univariate analysis revealed that the pT stage, pN stage, LNMR, and tumor differentiation were associated with regional lymphatic recurrence. A multivariate analysis revealed that the LNMR and tumor differentiation are independent risk factors for regional lymphatic recurrence. Other clinical and histological parameters (e.g., gender, tumor location, pathological stage, surgical procedure, and adjuvant chemotherapy) were not significant risk factors, as shown in Table 3.

**Table 3:** Univariate and multivariate analyses of the risk factors related to nodal recurrences.

Variables	Univariate analysis			Multivariate analysis		
	HR	95% CIs	P	HR	95% CIs	P
Age	1.231	0.900–1.683	0.194	1.129	0.821–1.552	0.455
<60 vs. ≥60						
Gender	1.142	0.728–1.791	0.564	1.378	0.862–2.204	0.181
Male vs. female						
LNMR	20.125	7.836–51.687	<0.0001	6.06	1.162–30.59	0.03
Tumor differentiation	1.252	1.095–1.430	0.001	1.165	1.012–1.341	0.03
Well vs. moderately vs. poorly						
pT stage	1.308	1.083–1.581	0.005	1.181	0.955–1.461	0.125
T1–2 vs. T3–4						
pN stage	1.640	1.394–1.930	<0.0001	1.320	1.000–1.745	0.051
N0 vs. N1 vs. N2 vs. N3						
Location of tumor	0.848	0.662–1.088	0.195	0.860	0.654–1.132	0.283
Upper vs. Middle vs. Lower						
Lymphadenectomy	0.700	0.477–1.028	0.069	0.862	0.567–1.311	0.487
Three-field vs. two-field						
Adjuvant chemotherapy	1.311	0.960–1.790	0.088	0.786	0.537–1.151	0.216
Yes vs. No						

Upper: Upper thoracic esophagus; Middle: Middle thoracic esophagus; Lower: Lower thoracic esophagus.

## Discussion

The results of this study demonstrated that approximately half of patients with esophageal SCC experienced treatment failure during the observation period. Locoregional recurrence (especially single-station lymph node recurrence) was the most common type of failure. Neck/supraclavicular (61 of 207 cases, 29.5%) and mediastinal lymph nodes (97 of 207 cases, 46.7%) showed the highest recurrent rates of all locations after the diagnosis of esophageal SCC and resection treatment. Our results approximated those of previously published studies [13], [14]. The locoregional recurrence rate was reported to be 30% for radical resection and 60% for R1 or R2 resections. Chen et al. [3] reported that the recurrence pattern was locoregional in 52 of 96 patients who received esophagectomies with two-field lymph node dissection (primarily mediastinal in 41 and single cervical/supraclavicular in eight). A study of 171 patients who underwent radical esophagectomies and three-field dissections revealed that 35 patients developed locoregional recurrences; among these patients, 27 experienced a recurrence in only one site [2]. The strength of the current study is that it contains the largest sample size of patients who experienced treatment failure after a curative surgery.

In our clinical practice, the overall survival rate of patients with locoregional recurrence was worse. Despite the controversy regarding whether PORT improves survival for all patients, previous studies have reported potential benefits. Chen et al. [8] reported that treatment with PORT reduces the likelihood of cervical and mediastinal recurrence by more than 50%, which is consistent with the findings of Xiao et al. [7]. Previous reports suggest that PORT is justified for patients who are at a high risk of locoregional recurrence (e.g., those with T3–4 tumors, node-positive disease, or close/positive margins). However, three published randomized trials have reported that survival benefits are not associated with the addition of PORT, although one of them revealed local failure was reduced in the PORT arm [15], [16], [17]. Possible explanations for this result are that they did not stratify the patients based on their stage and the sample size was not large enough to detect an improvement in survival. Another reason could be the increased mortality in the radiation treatment arm, which resulted in radiation fibrosis of the lung, noncancerous pericardial and pleural effusion, and alimentary tract hemorrhage. These outcomes often occur during and after irradiation with two-dimensional treatment planning using the simple AP-PA techniques common when those studies were conducted in the late 1980s and early 1990s. The radiation techniques currently performed using three-dimensional conformal RT (3D-CRT) planning with daily image guidance perform better with regard to protecting normal tissues and improving the accuracy of irradiation. However, prospective data is needed to confirm the value of PORT based on 3D-CRT techniques.

Furthermore, the optimal CTV design to be used for 3D-CRT planning remains under investigation, and no clear consensus exists concerning the extent of the PORT CTV to treat radically resected esophageal SCC. The esophageal submucosa has an extensive lymphatic vertical distribution, which is the anatomical basis of lymph node metastasis in esophageal cancer. Numerous studies have suggested that multiple level and skipped node metastases are commonly observed in esophageal SCC [18], [19], [20]. Therefore, the standard CTV of postoperative prophylactic radiotherapy should include the esophageal tumor bed as well as the supraclavicular, mediastinal, and upper abdominal areas. However, the irradiation range differed across various studies that included (1) the bilateral supraclavicular areas and the entire mediastinum [15]; (2) the bilateral supraclavicular areas, the entire mediastinum, and the left gastric lymph nodes [21]; (3) the tumor bed alone [16]; and (4) a T-shaped field including the bilateral lower cervical and supraclavicular areas as well as the upper portion of the mediastinum [7]. Although the issue of which lymph node regions to include in the CTV is controversial, we believe that the patterns of treatment failure after surgery can provide additional guidance in establishing the CTV. Our data suggest that recurrences in the bilateral supraclavicular areas and the superior mediastinum are more frequent than in other regions and that a CTV consisting of the bilateral supraclavicular and superior mediastinal areas (rather than all lymphatic drainage regions) would be adequate for the vast majority of patients. Qiao et al. [21] studied 102 patients who underwent PORT after radical resection for esophageal SCC (T3/4 or N1) and found that the use of a regional portal is not associated with compromised survival rates compared with the use of extensive portal RT. Lu et al. [22] retrospectively assessed the survival data of 204 patients and reported that irradiation of the left gastric area is unnecessary after radical surgery when the primary tumor site is in the upper, middle, or middle-upper thirds of the thoracic esophagus; similarly, irradiating the bilateral supraclavicular area is unnecessary when the primary site is in the lower and middle lower thirds. Huang et al. [20] argued that a selective regional irradiation including the correlated lymphatic drainage regions should be performed based on clinical and pathological risk factors such as pT stage, tumor length and histological differentiation (odds ratios of 1.145, 1.501, and 1.973, respectively). Prospective randomized trials should be undertaken to further validate irradiation CTVs. Our hospital is currently conducting a prospective investigation of the optimal CTV delineation for PORT, and the results will be reported in a separate analysis.

Anastomotic recurrence was not common in our sample (10.1% of those who relapsed) though patients with stage pT3-4a cancer were common. Nakagawa et al. [2] reported that of the 30 patients they examined who experienced locoregional recurrence, 2.9% appeared in the anastomotic stoma. This value was 7.9% in Chen's study [16]. Given that tumor invasion increases the difficulty of complete resection, we hypothesize that these patients have a higher risk of developing anastomotic recurrences. Previous studies have suggested that extended-volume external-beam radiotherapy that encompasses the tumor bed and the anastomotic site is feasible and safe for patients with high-risk T3-4 esophageal cancer after esophagectomy [23]. Nevertheless, determining whether both the anastomotic site and the tumor bed required irradiation was difficult because the recurrence rate was not high.

Multiple studies have reported that depth of invasion, differentiation, number of lymph nodes with positive metastases, and lesion length are correlated with locoregional recurrence after esophagectomy [1], [2]. The current study investigated whether the LNMR and differentiation were associated with disease recurrence. Bhansali et al. [24] and Kimura et al. [25] reported that the number of resected positive nodes was correlated with local-regional recurrence and the survival of patients with thoracic esophageal carcinoma; this result is consistent with our findings, which suggests that lymph node recurrence increases dramatically with a higher LNMR after radical surgery. We were unable to identify whether extensive portal radiotherapy or systematic therapy is more effective or which should be the preferred treatment for these patients.

This study has several potential limitations. First, to investigate the definitive locations of recurrence and metastasis, the selection of enrolled patients depended on positive CT scans taken during the follow-up period. This inclusion criterion censored patients who were lost to follow up, relapse-free, or who had died from other diseases, which might have led to an inherent bias. Second, our conclusion concerning the CTV of PORT was obtained based on the initial regional recurrence of patients without observing a full natural death period. In addition, the presence or absence of failure was only proven histopathologically in a fraction of patients. This lack of data might underestimate the risk of recurrence occurring outside the proposed CTV. Finally, approximately half of the patients in this study received adjuvant chemotherapy.In general, adjuvant chemotherapy may be considered for node positive patients, although there is little data to support its use in these cases [26]. Our data showed that adjuvant chemotherapy did not correlate with nodal recurrences, but whether adjuvant chemotherapy affects tumor control remains unclear.

## Conclusions

In summary, locoregional recurrence (especially single-station lymph node recurrence) was the most common type of recurrence after surgery among patients diagnosed with esophageal SCC. Neck/supraclavicular and mediastinal lymph nodes had the highest recurrence rates among patients treated via surgical resection. Most of the lymphatic recurrences experienced by the patients in this study would have been covered by the proposed CTV. However, the potential value of PORT and the extent of the PORT CTV must be determined in further prospective investigations.
